# Advantages of implantology-related preventive infra-occluded primary teeth extraction in cases of underlying agenesis: A retrospective study of 290 patients

**DOI:** 10.4317/medoral.27675

**Published:** 2025-10-17

**Authors:** Adrien Leporcq, Romain Nicot, Gwenael Raoul, Yamen Komakli, Ludovic Lauwers

**Affiliations:** 1 Univ. Lille, CHU Lille, Department of Oral and Maxillofacial Surgery, F-59000 Lille, France; 2 Univ. Lille, CHU Lille, INSERM, Department of Oral and Maxillofacial Surgery, U1008 - Advanced Drug Delivery Systems, F-59000 Lille, France; 3 Univ. Lille, CHU Lille, Department of Oral and Maxillofacial surgery, URL 2694 - METRICS, F-59000 Lille, France

## Abstract

**Background:**

This study aimed to evaluate the impact of preventive infra-occluded primary teeth extraction in patients with dental agenesis on the success of pre-implantation and implantation treatment, and the preservation of alveolar ridge height.

**Material and Methods:**

This retrospective analysis included 290 patients with dental agenesis treated at Lille University Hospital between December 2019 and January 2024, including 92 patients with infra-occluded primary teeth. Clinical and radiographic data were collected, and the implant outcomes were compared between those who underwent preventive versus delayed extractions.

**Results:**

Preventive extraction was associated with 100% preservation of alveolar ridge height and eliminated the need for vertical bone grafting. In contrast, 28% of patients with delayed extractions experienced vertical bone loss and implant infraposition despite complex grafting procedures. The second primary molars were the most frequently infra-occluded (66.5%), mostly at a moderate degree. The median ages at diagnosis, extraction, and implantation were 14, 17, and 18 years, respectively.

**Conclusions:**

Compared to delayed extraction, preventive extraction of infra-occluded teeth in agenesis cases facilitates better implant positioning and reduces the need for complex pre-implant surgeries. Timely intervention is key, particularly in the maxilla, to avoid vertical bone loss and implant complications.

## Introduction

Dental agenesis is a congenital number-of-teeth anomaly, mainly observed in the permanent dentition. It is characterized by the absence of development of one or more teeth and can have considerable functional, aesthetic, and psychological repercussions (1). Its prevalence varies between 1.6% and 9.6%, depending on the study (2). There are several forms of agenesis: hypodontia, characterized by the absence of 1-5 teeth; oligodontia, characterized by the absence of at least six teeth; anodontia, characterized by a total absence of teeth (2 , 3). Oligodontia and anodontia are rare, affecting around 0.14% of the population, and are frequently associated with syndromes of ectodermal origin (3 , 4). Dental agenesia treatment may consist of closing the edentulous spaces or maintaining them using dental prostheses, depending on the face type, the dentoskeletal disorder, and the number of agenesia (5).

Infra-occlusion of primary teeth in relation to the occlusal plane is a frequently encountered clinical phenomenon. In these cases, teeth invaginate into the alveolar bone is often accompanied by tilting of adjacent teeth and egression of antagonistic teeth, with reduced arch length (6 - 9). The mechanism of this phenomenon remains poorly understood, although the hypothesis of a stoppage in vertical growth linked to ankylosis of the periodontal ligament is frequently put forward (10). Infra-occlusion can occur with or without underlying tooth germ and is often associated with delayed alveolar growth (11 - 13). The preservation and restoration of infra-occlusal teeth in cases of agenesis are frequently favored in order to maintain a stable occlusion and promote harmonious growth of the masticatory apparatus (5). However, no studies have investigated the impact of these infra-occluded teeth on implant therapy (14 - 18).

The main aim of this study was to analyze the management of pre-implantation and implantation treatments in patients with dental agenesis and to assess the benefits of preventive extraction of infra-occluded primary teeth to reduce the need for complex pre-implant reconstructions. The study's secondary objective was to describe the specific clinical characteristics of this patient population.

## Material and Methods

This retrospective observational study was conducted in the dental agenesis consultation unit of the North of France O-Rares Competence Centre, the Oral and Maxillo-Facial Surgery Department, Lille University Hospital, a center specializing in the care, diagnosis, monitoring, and research of rare diseases. The center is approved by the health authorities and works in conjunction with a reference center.

The study included patients with at least one dental agenesis who attended a specialized agenesis consultation between December 2019 and January 2024. The absence of third permanent molars was not considered agenesis. Patients who had not received implant treatment, situations of complete edentulism not allowing evaluation of occlusal parameters and cases of overall failure of implant treatment were excluded for analysis of pre-implant treatment and the impact of early extraction.

Data were retrospectively extracted from medical records by 1 expert examiner in the field. A review by another expert examiner was carried out without the presence of the first examiner. In the event of disagreement, a joint agreement was reached between the 2 examiners. A data processing declaration certificate was issued by the Lille University Hospital data protection officer under reference MR 004. This study followed the Declaration of Helsinki on medical protocols and ethics.

**Figure 1:**
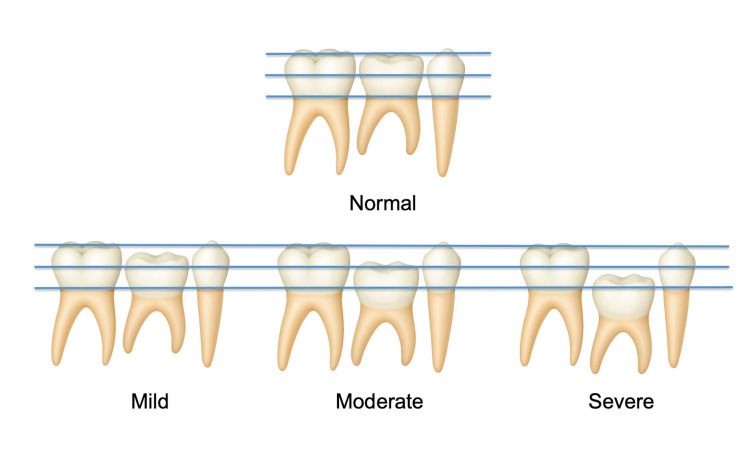
Illustration of dental infra-occlusions classification.

Approval was obtained from the Ethics Committee of the French Society of Stomatology, Maxillofacial Surgery and Oral Surgery, with approval number CEth-SFSCMFCO 002/2024. Demographic, clinical, and radiological data were collected. For the entire cohort, the variables studied were gender and number and location of agenesis. For patients with infra-occlusion, the variables studied also included degree of infra-occlusion, age at diagnosis, age at extraction, age at pre-implantation treatment, age at implant placement, management of infra-occlusions in orthodontic treatment, type of pre-implantation treatment (vertical, horizontal, sinus lifting, nerve bypass), and implant references and positions. These data were obtained from panoramic radiographs, medical observations, operative reports, and consultation letters. The degree of infra-occlusion was assessed radiologically on the last panoramic radiograph available and classified as mild, moderate, or severe (Figure 1). Mild was considered when the occlusal table was 1mm below the occlusal plane; moderate was considered when the occlusal table was level with the contact point of the adjacent tooth; severe was considered when the occlusal table was below the contact point (5 , 19).

1

## Results

The study included 290 patients with dental agenesis, nine with hypodontia, 276 with oligodontia, and five with anodontia. The mean number of agenesis observed was 11.00±5.33. The cohort included 161 females and 129 males.

Analysis of the medical records identified 269 infra-occluded teeth in 92 patients with at least one infra-occlusion in relation to tooth agenesis, 119 in females and 150 in males. The average infra-occlusion was 0.93±1.64. Of the patients with no infra-occlusion, 59% (CI 52-66) were female and 41% (CI 43-48) were male. Among patients with at least one infra-occlusion, 52% (CI 42-62) were male and 48% (CI 38-58) were female.

**Figure 2:**
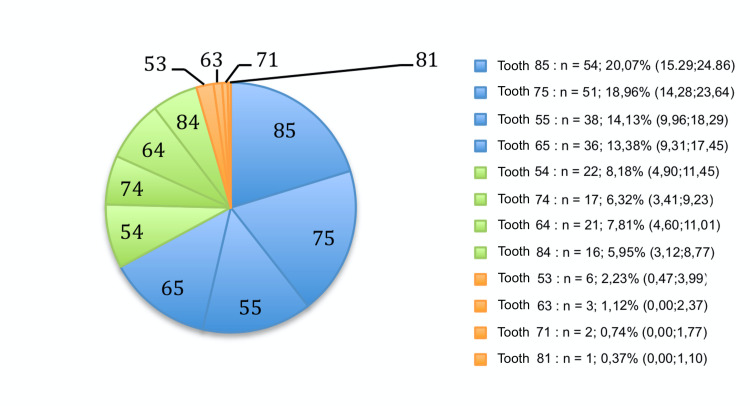
Distribution of infra-inclusions by dental site.

The most frequently infra-occluded teeth were the second primary molars (66.5%) (Figure 2). In fact, 20.1% (CI 15.3-24.9) concerned tooth 85, 19.0% (CI 14.3-23.6) concerned tooth 75, 14.1% (CI 10.0-18.3) concerned tooth 55, and 13.4% (CI 9.3-17.5) concerned tooth 65. The first primary molars were the second most frequently infra-occluded (28.3%), including 8.2% (CI 4.9-11.5) in tooth 54, 7.8% (CI 4.6-11.0) in tooth 64, 6.3% (CI 3.4-9.2) in tooth 74, and 6.0% (CI 3.1-8.8) in tooth 84. The other teeth were very rarely affected by infra-occlusions and fluctuated between 0.4% and 2.2%, depending on the area. Moderate infra-occlusion was the most observed type in males and females, at 44.2% (CI 38.3-50.2), followed by mild (30.5%; CI 25.0-36.0) and severe (25.3%; CI 20.1-30.5).

2

Most primary first molars showed a severe degree of infra-occlusion, as did tooth 54 (40.9%; CI 20.4-61.5), tooth 64 (42.9%; CI 21.7-64.0), and tooth 74 (47.1%; CI 23.3-70.8). Tooth 84 was an exception, as most infra-occlusions in it were moderate (56.3%; CI31.9-80.6). A moderate degree of infra-occlusion was also common in the primary second molars, as did tooth 55 (42.1%; CI 26.4-57.8), tooth 65 (36.1%; CI 20.4-51.8), tooth 75 (47.1%; CI 33.4-60.8), and tooth 85 (57.4%; CI 44.2-70.6) (Table 1).

Table 1

The mean age at infra-occlusion diagnosis was 14.9±6.0 years, with a median of 14 years. The mean age at extraction of the infra-occluded tooth was 18.1±6.1 years, with a median of 17 years. The mean age at implantation was 20.9±6.7 years, with a median of 18 years.

**Figure 3:**
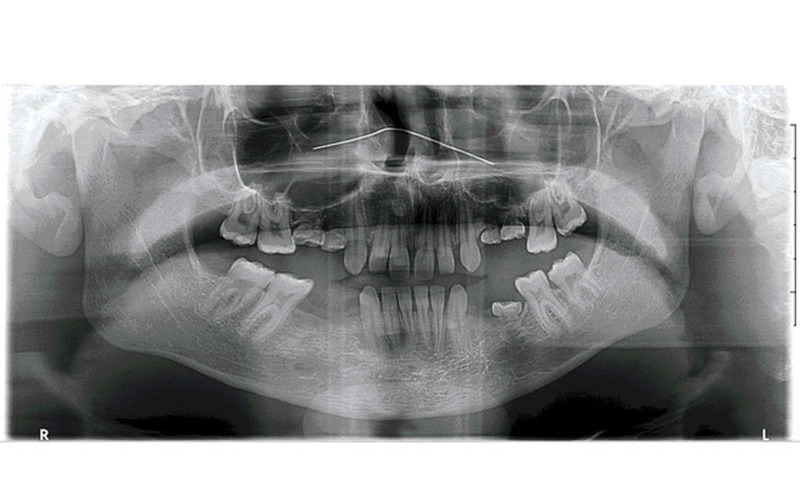
Example of severe maxillary and mandibular infra-occlusions.

**Figure 4:**
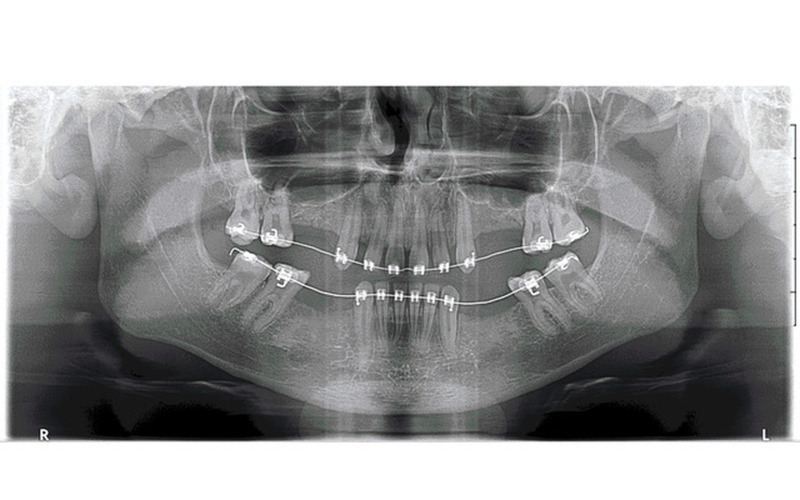
Example of preventive management of severe maxillary and mandibular infra-occlusions (after dental extractions and before dental implants). No complex vertical bone grafts is required to achieve the correct vertical position of the implants.

Of the 92 patients with at least one infra-occlusion, 46 had been implanted by the time of data analysis. Two patients were excluded from the analysis, one because of overall failure and the other because of complete edentulism that made analysis impossible. A review of the medical records showed that 26 patients had preventive extractions, and 18 had delayed extractions. All patients in the preventive extraction group had preservation of alveolar ridge height, regardless of the infra-occlusion degree (Figure 3 and Figure 4). No vertical grafting of the alveolar ridge was required. The implant positions were consistent with those of the adjacent teeth, and some grafting, mainly horizontal or sub-sinusal, was sometimes performed. Conversely, five patients (28%) who underwent delayed extractions had a loss of the alveolar ridge height with implants in an infra-position with respect to the adjacent teeth, despite occasionally undergoing complex vertical bone grafting of the alveolar ridges, independently of the degree of infra-occlusion. The remaining 14 patients (72%) had no vertical bone loss and had compliant implant positions. Of these, 15% had a compliant implant level due to successful complex vertical bone grafting from the alveolar ridges in cases of severe damage, 8% due to slight management of the infra-occluded teeth in the multi-bracket orthodontic appliance (orthodontic extrusion), and 77% could not be explained. Of the unexplained vertical preservation, 70% were concerned with late extractions in the mandible for moderate or severe infra-occlusion, while 30% were concerned with late extractions in the maxilla for slight infra-occlusion.

3

4

Conventional implants from Nobel Biocare (Kloten, Switzerland) or Anthogyr (Sallanches, France) were used in all implantation sites. The length was 8mm in 11.7% of the implants, 10mm in 43.6%, 11.5mm in 14.9%, 12mm in 11.7%, 13mm in 9.6%, and 15mm in 4.3%. The diameter was narrow (3.4-3.75mm) in 48.9% of the implants and regular (4.0-4.6mm) in 51.1%. No very narrow or wide implants were placed.

## Discussion

This study showed that the choice between preventive and delayed extraction significantly impacted the use of complex pre-implant surgery and the success of implant positioning in relation to adjacent teeth. Complex pre-implant surgery aims to reconstruct a three-dimensional bone site where the alveolar ridge undergoes vertical and horizontal resorption. However, these pre-implant treatments, which aim to restore the bone structure in the vertical and horizontal directions, often result in significant vertical resorption that could lead to implant infra-position. Other, less complex, pre-implant treatments, including horizontal bone apposition and sub-sinus filling, are commonly used but do not enable vertical bone crest reconstruction, leading to implant sub-position and larger crowns. These less complex approaches are used as complement treatments if verticality is preserved.

A large sample of 290 patients with agenesis, with a high proportion of oligodontia, reinforces the reliability of our results. The gender distribution showed a female predominance in agenesis, which is consistent with previous studies (2 , 20). In the absence of infra-occlusion, this female predominance was even more pronounced. On the other hand, in the case of infra-occlusion, the distribution between the genders was reversed, with a male predominance in agenesis and a greater number of infra-occlusions in males.

Infra-occlusion of primary teeth was identified in 31.7% of the patients. Most infra-occluded teeth were primary molars, more the second than the first. This trend aligns with other studies that showed a more frequent infra-occlusion of primary molars than other teeth (21 , 22). Medium infra-occlusions were most frequent, followed by slight and severe infra-occlusions. Most primary second molars were moderately affected, whereas most primary first molars were severely affected, requiring more complex implant management.

The median age at infra-occlusion diagnosis (14 years) aligns with the growth period. However, it could be even earlier if patients were referred earlier. The median age at infra-occluded tooth extraction (17 years) was later than the median age at diagnosis. Extractions were carried out on average three years after diagnosis, which may have accentuated the degree of infra-occlusion and increased the complexity of implant rehabilitation. This delay was partly due to the reluctance of patients and practitioners to extract teeth. The median age at implantation (18 years) aligns with recommendations for implant placement after bone maturation to optimize results.

In the patients treated with preventive extractions, preservation of alveolar ridge height without complex pre-implant treatments was systematically achieved, irrespective of the degree of infra-occlusion, ensuring a conformal implant position in relation to the adjacent teeth. This finding underlines the importance of preventive avulsion in maintaining vertical bone stability and avoiding the need for complex vertical pre-implant treatments. Although some bone grafting, mainly horizontal or sub-sinusal, was necessary, its frequency was comparable to that observed in the general implant population.

In the group of patients treated with late extractions, 43% showed a loss of the alveolar ridge height. This loss was successfully restored by complex pre-implant treatment in 15%, while restoration was unsuccessful in 28% despite the sometimes complex pre-implant treatment. Furthermore, a large proportion of patients treated with late extractions maintained their alveolar ridge height without pre-implant treatments. This could be explained by the orthodontic extrusion of infra-occluded teeth by including them in the multi-bracket appliance. In some cases, this made it possible to correct certain mild infra-occlusions. However, their inclusion in this study was far too limited and concerned only mild degrees. Larger-scale studies covering all degrees of infra-occlusion are needed. However, it would be difficult to support this theory if infra-occlusion resulted from ankylosis. For other cases, no explanation has been found. The results indicated that late extractions with vertical bone preservation were found more frequently in the mandible, especially for moderate and severe involvement, than the maxilla, where these vertical preservations mainly concerned mild infra-occlusions. However, the question arises whether a mild infra-occlusion, accompanied by a conforming bone height, should be considered a true infra-occlusion. In this context, orthodontic extrusions are not performed on real infra-occlusions, and maxillary vertical bone preservation in cases of delayed avulsions is non-existent. Natural vertical bone preservation in late management would only be in the mandibula, while it is important to perform preventive maxillary avulsion to avoid implant infra-positions and complex pre-implant reconstructions.

This study had several limitations. First, it is a descriptive retrospective study that might have potential biases related to data quality and completeness. The interpretation and performance of cases are team-dependent. Although the sample was relatively large, only half of the patients have been implanted to date. A new study on this same sample in a few years could be interesting. It would allow for a larger number of implanted patients, thus increasing the study's power.

## Conclusions

Infra-occlusion of primary teeth in patients with dental agenesis is a relatively frequent phenomenon, with significant repercussions on orthodontic and implant management. The results of this study suggest that preventive extractions allow for better maintenance of alveolar ridge height and implant positioning than late extractions while reducing the need for complex pre-implant treatments. Conversely, late extractions present an increased risk of complications, including loss of alveolar bone height and implant infrapositioning, particularly in the maxillary lateral sectors, despite using complex vertical pre-implant treatments. Orthodontic extrusion of infra-occluded teeth by including them in the multi-bracket appliance has made it possible in some cases to correct some infra-occlusions. This possibility should be considered as soon as possible. These factors underline the importance of choosing the right time to perform extraction, depending on the degree of infra-occlusion and the location of the damage, in order to optimize results. Future larger-scale studies are needed to expand on these observations. In addition, it would be useful to compare our results with those of similar studies.

## Figures and Tables

**Table 1:** Table Table of dental infra-occlusions by localization and degree.

Infra-occlusion degrees	Dental localization	52	62	53	63	54	64	55	65	71	81	74	84	75	85	Total
Mild		0	1	3	2	6	6	12	14	1	1	2	3	15	16	82
Moderate		1	0	3	1	7	6	16	13	1	0	7	9	24	31	119
Severe		0	0	0	0	9	9	10	9	0	0	8	4	12	7	68
Total		1	1	6	3	22	21	38	36	2	1	17	16	51	54	269

1

## Data Availability

Declared none.

## References

[1] Kotecha S, Turner PJ, Dietrich T, Dhopatkar A (2013). The impact of tooth agenesis on oral health-related quality of life in children. J Orthod.

[2] Polder BJ, Van’t Hof MA, Van der Linden FPGM, Kuijpers-Jagtman AM (2004). A meta-analysis of the prevalence of dental agenesis of permanent teeth. Community Dent Oral Epidemiol.

[3] De La Dure-Molla M, Fournier BP, Manzanares MC, Acevedo AC, Hennekam RC, Friedlander L (2019). Elements of morphology: standard terminology for the teeth and classifying genetic dental disorders. Am J Med Genet A.

[4] Gill DS, Barker CS (2015). The multidisciplinary management of hypodontia: a team approach. Br Dent J.

[5] Medio M, De la Dure Molla M (2014). Treatment of infra-occluded primary molars in patients with dental ageneses. Int Orthod.

[6] Noble J, Karaiskos N, Wiltshire WA (2007). Diagnosis and management of the infraerupted primary molar. Br Dent J.

[7] Ekim SL, Hatibovic-Kofman S (2001). A treatment decision-making model for infraoccluded primary molars. Int J Paediatr Dent.

[8] Hua L, Thomas M, Bhatia S, Bowkett A, Merrett S (2019). To extract or not to extract? Management of infraoccluded second primary molars without successors. Br Dent J.

[9] Akgöl BB, Üstün N, Bayram M (2024). Characterizing infraocclusion in primary molars: prevalence, accompanying findings, and infraocclusion severity and treatment implications. BMC Oral Health.

[10] Kurol J (1984). Infraocclusion of primary molars. An epidemiological, familial, longitudinal clinical and histological study. Swed Dent J Suppl.

[11] Kurol J, Thilander B (1984). Infraocclusion of primary molars with aplasia of the permanent successor: a longitudinal study. Angle Orthod.

[12] Baccetti T (1998). A controlled study of associated dental anomalies. Angle Orthod.

[13] Kula K, Tatum BM, Owen D, Smith RJ, Rule J (1984). An occlusal and cephalometric study of children with ankylosis of primary molars. J Pedod.

[14] Lauwers L, Raoul G, Lauwers R, Antunes D, Bovis M, Nicot R (2023). Pre-implant and implant management of oligodontia patients: a 10-year retrospective study. J Stomatol Oral Maxillofac Surg.

[15] Lauwers L, Raoul G, Nicot R (2024). Pre-implant surgery complexity for achieving implant-supported prosthetic rehabilitation in oligodontia patients: a retrospective study. BMC Oral Health.

[16] Lauwers L, Wojcik T, Delbarre A, Movaghar R, Ferri J (2009). [Hypodontia: therapeutic strategy elaborated from 30 cases]. Rev Stomatol Chir Maxillofac.

[17] Thuaire A, Nicot R, Raoul G, Lauwers L (2023). Surgical bone augmentation procedures for oral rehabilitation of patients with oligodontia: a review with a systematic approach. J Stomatol Oral Maxillofac Surg.

[18] Laventure A, Raoul G, Nicot R, Ferri J, Lauwers L (2021). Staged autogenous calvarial bone grafting and dental implants placement in the management of oligodontia: a retrospective study of 20 patients over a 12-year period. Int J Oral Maxillofac Surg.

[19] Brearley LJ, McKibben DH (1973). Ankylosis of primary molar teeth. I. Prevalence and characteristics. ASDC J Dent Child.

[20] Re D, Clivio A, Butti AC, Nobili A, Mulè G (2023). Evaluation of the prevalence of dental agenesis through the use of orthopantomography in a sample of subjects residing in Lombardy and Piedmont regions. Eur J Paediatr Dent.

[B21] Bergendal B (2008). When should we extract deciduous teeth and place implants in young individuals with tooth agenesis?. J Oral Rehabil.

[22] Nordquist I, Lennartsson B, Paulander J (2005). Primary teeth in adults--a pilot study. Swed Dent J.

